# Inter‐Observer Processing and Measurement Error Are Low for 2D Dental Measurements on Shared microCT Scans

**DOI:** 10.1002/ajpa.70001

**Published:** 2025-01-31

**Authors:** Kaita N. Gurian, Debra Guatelli‐Steinberg, W. Scott McGraw, Jess Rychel, Mackie C. O'Hara

**Affiliations:** ^1^ Department of Anthropology Ohio State University Columbus Ohio USA; ^2^ School of Medicine West Virginia University Morgantown West Virginia USA; ^3^ Department of Sociology Purdue University West Lafayette Indiana USA

**Keywords:** enamel thickness, inter‐observer error, microCT

## Abstract

**Objectives:**

Sharing micro‐computed tomographic (μCT) scans of teeth increases data accessibility and reduces the need for repeated scans of any given specimen. However, the use of the same TIFF stacks or DICOMs by multiple individuals has the potential to introduce new sources of error. Here, we explore whether use of the same μCT scans by different persons produces comparable results.

**Materials and Methods:**

Worn (*N* = 11) and unworn (*N* = 4) *Cercocebus atys* upper molars (UM1 *N* = 8, UM2 *N* = 7) were μCT scanned using a Bruker Skyscan 1172 High Resolution Ex Vivo Scanner at a resolution of 22 μm. Two individuals (K.N.G. and M.C.O.) created a 2D mesial slice for each TIFF stack (tooth). Worn teeth were reconstructed by K.N.G. and M.C.O. Three researchers (M.C.O., K.N.G., and J.R.) measured tooth shape, linear enamel thickness, average enamel thickness, and relative enamel thickness (AET and RET). Inter‐observer percent error was calculated for each measurement. Univariate ANOVAs were calculated to evaluate variance due to slice maker, reconstructor, tooth, and measurer when percent error averaged > 5%.

**Results:**

For unworn teeth, error was generally low and largely due to the person doing the measurement. For worn teeth, wear reconstructor was a statistically significant source of variation for AET and RET.

**Discussion:**

We found that (1) inter‐observer error was generally low, (2) linear measurements are prone to error, (3) worn teeth did not present an additional source of error as compared to unworn teeth, and (4) different people can use the same μCT scans to reliably reconstruct, slice, and measure teeth.

**Summary**

- We test whether different people using the same μCT scans produce comparable results.
- Results show that different persons can use the same μCT scans to reliably reconstruct, slice, and measure teeth.

## 1 Introduction

The recent practice of sharing micro‐computed tomographic (μCT) scans of teeth increases data accessibility and reduces the need for repeated scans of any given specimen. However, the use of the same TIFF stacks or DICOMs by multiple individuals for a single research purpose (e.g., enamel thickness measurements) has the potential to introduce new sources of inter‐observer error in measurements because slight variation in plane positioning created by different people making different 2D slices of the same μCT scan can affect the shape of the slice created, which can impact cusp reconstruction and all subsequent measurements (Benazzi et al. 2014; O'Hara and Guatelli‐Steinberg 2022). μCT scans can also be costly and time‐intensive, with scans often being charged by the hour. Sharing μCT scans reduces the need for individual researchers to invest in μCT scanners or in importing physical materials (and the necessary permissions needed) to scan. However, to share scans, dental researchers need to be assured that multiple persons using the same μCT scans can produce comparable results. Here, we consider the magnitude and impact of inter‐observer error at four moments: (1) the tooth in question, (2) 2D slice position, (3) cusp reconstruction, and (4) measurement of tissues.

The use of μCT scans is well established in dental anthropology (Bailey, Benazzi, and Hublin 2014; O'Hara and Guatelli‐Steinberg 2022; Skinner et al. 2008a, 2008b; Smith et al. 2009). μCT scans are non‐destructive, making them ideal for use in assessing fossil taxa or fragile specimens (Bailey, Benazzi, and Hublin 2014; Skinner et al. 2008b). Teeth, particularly fossilized ones, can be fragile, making limited handling desirable. Moreover, because it is becoming increasingly difficult to transport specimens, sharing μCT scans via online repositories such as MorphoSource (http://www.morphosource.org) can significantly expand access to these specimens. When digital versions of teeth are made available, accessibility is increased, researchers' travel costs and carbon footprint are reduced, and specimen safety is improved.

Digital dental anthropology methods also promote use of new research avenues in both 2D and 3D. Using μCT scans of teeth, researchers have compared the external and internal morphology of various extinct and extant taxa using crown and EDJ shape analysis in both 2D and 3D (Smith et al. 2006, 2009). μCT scans are also commonly used for applications of geometric morphometric analysis, enamel thickness measurements, and morphological comparisons in modern humans, fossil hominins, and primate dentitions (Benazzi et al. 2009; Smith et al. 2006; Skinner et al. 2008b). Enamel metrics are also important in reconstructing the dietary characteristics, taxonomy, and phylogeny of extant and extinct primate and hominoid species (Bailey, Benazzi, and Hublin 2014; Benazzi et al. 2014; Martin, Olejniczak, and Maas 2003).

Measuring enamel thickness on 2D virtual sections is commonly performed using μCT dental scans and involves creating a plane of section through anatomical points on tooth crowns (typically the mesial cusps for molars and a bisection labio‐lingually for anterior teeth). Standards have been established regarding the position of this plane, originally derived for histological sectioning (Benazzi et al. 2009, 2014; Martin, Olejniczak, and Maas 2003; Molnar and Gantt 1977). In many teeth, cusp tips are worn and require digital reconstruction before enamel thickness can be calculated. For a full description of the methods and requirements for reconstructing cusp tips, see O'Hara and Guatelli‐Steinberg 2022. Following cusp tip reconstruction (when necessary), measurements such as linear distance between EDJ and OES, enamel cap area, enamel dentine junction length, and dentine area are taken. These variables can then be used to calculate commonly used measures of enamel thickness such as average enamel thickness (AET) and relative enamel thickness (RET) (Martin 1985). At each procedural step, the researcher has the potential to influence the final measurements of enamel thickness either through conscious decision making, experience level, or random variation. For example, slight variation in plane positioning can affect the shape of the slice created, which can impact cusp reconstruction and all subsequent measurements (Benazzi et al. 2014; O'Hara and Guatelli‐Steinberg 2022).

In this study, we examine whether there are significant inter‐observer differences in 2D measurements of dental tissues. We aim to (1) determine if there are significant inter‐observer differences in 2D measurements of dental tissues from different 2D slices and reconstructions, and (2) identify whether, for 2D measurements with significant inter‐observer differences (> 5% average error), the main source of measurement variation occurs at the production of the 2D slice, the reconstruction of the cusp (for worn teeth), or the observer taking the measurement. We also consider the role that wear plays in error generation.

## 2 Materials and Methods

### 2.1 Samples

The study sample consists of 15 *Cercocebus atys* (sooty mangabey) upper molars (UM1 *N* = 8, UM2 *N* = 7) collected from naturally decedent individuals from the Taï National Park in Côte d'Ivoire (Table 1). All teeth were μCT scanned using a Bruker Skyscan 1172 High Resolution Ex Vivo Scanner at a resolution of 22 μm. Outputs were saved as TIFF stacks. Eleven teeth had significantly occlusal surface wear and necessitated digitally reconstructed cusp tips (UM1 *n* = 7, UM2 *n* = 4). μCT scans were processed using NRecon V1.7.4.2 to reduce noise and TIFF stacks were generated.

**TABLE 1:** Tooth types and specimen numbers used in this study grouped by wear level. Wear determined as categorized in Molnar (1971).

Tooth type	Unworn	Molnar stage 3	Molnar stage 4
UM1	TF 2020	16‐5	TF 24‐3, TF 94‐25, 2041, TF 22‐46, TF 2019, TF 2001
UM2	16‐5, TF 94‐25, TF 22‐46	TF 2108, 2010‐2	2016, 2229

**FIGURE 1:**
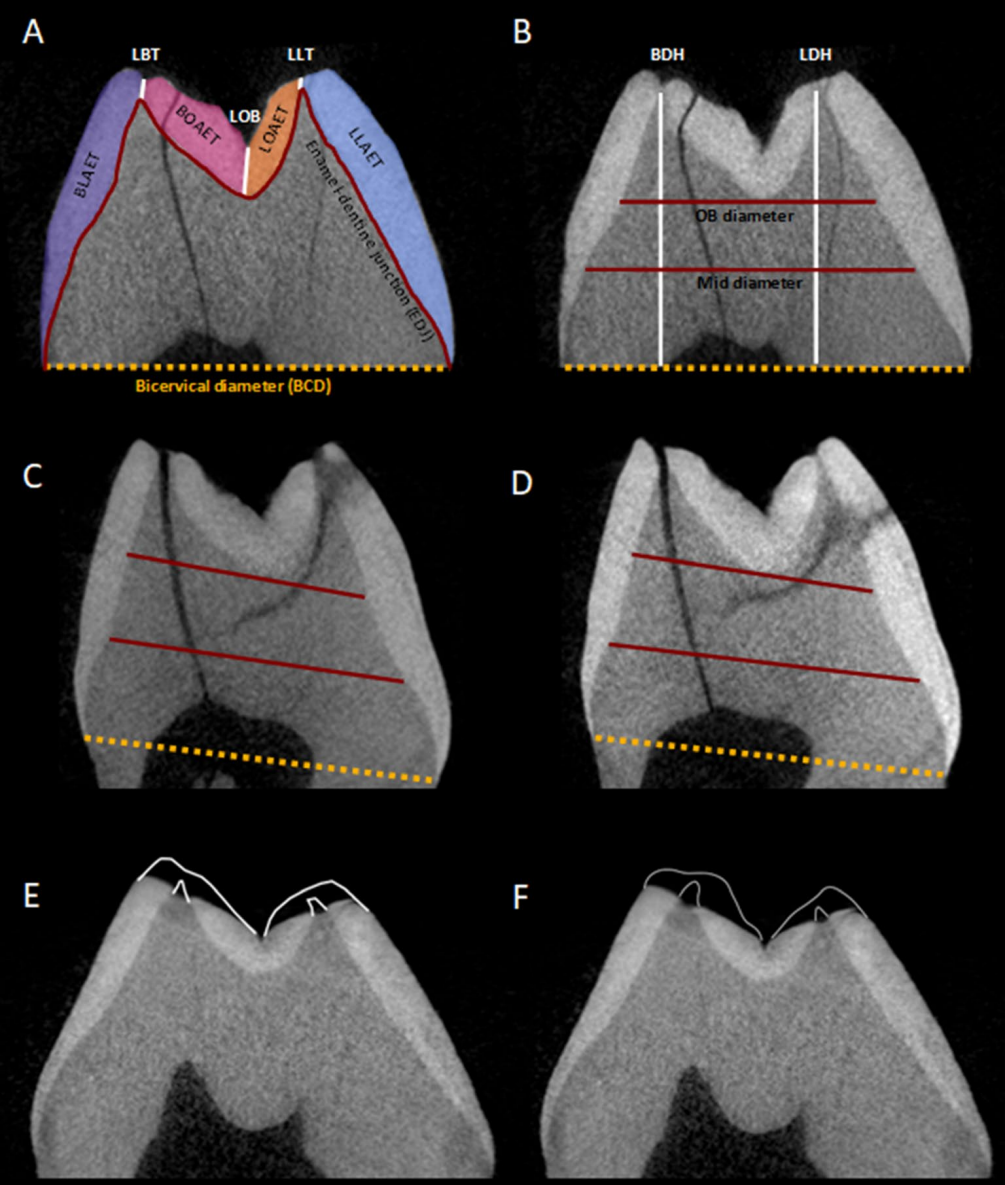
(A, B) All measurements taken on unworn and unreconstructed teeth shown on *C. atys* UM2 94‐7 slice made by M.C.O. Descriptions of measurements can be found in Table 2. (C, D) *C. atys* 94‐25 UM2 2D slice made by K.N.G. (C) and M.C.O. (D); dentine diameters and BCD are marked in red and yellow as in (A, B. E, F) *C. atys* 16‐5 UM1 slice made by K.N.G., reconstructed by M.C.O. (E) and K.N.G. (F) using the profile method outlined by O'Hara and Guatelli‐Steinberg (2022).

For each tooth, two individuals (K.N.G. and M.C.O.) created 2D mesial slices from the TIFF stack. If the tooth was worn, the cusp tip was reconstructed by K.N.G. and M.C.O. according to protocols set by O'Hara and Guatelli‐Steinberg (2022). Wear was defined as the dentine horn being exposed on both cusps, all worn teeth had both cusps worn and reconstructed, all unworn teeth had both unworn cusps and no reconstruction. O'Hara and Guatelli‐Steinberg define this as “extensive” wear, roughly corresponding to Molnar's stages 3–4 (Molnar 1971). All worn teeth in our sample corresponded to Molnar's stages 3 or 4 (Table 1). Since all worn teeth had similar levels of wear to O'Hara and Guatelli‐Steinberg's “extensive” wear category, we divided the sample into worn and unworn categories for analysis. Three researchers (M.C.O., K.N.G., and J.R.) measured tooth shape, linear enamel thickness, and AET on each of the two produced slices. Figure 1 and Table 2 illustrate and explain each measurement.

**TABLE 2:** Measurements taken on 2D slices, all measurements taken in mm.

Measurement	Abbreviation	Description	Application
2D shape			
Bicervical diameter	BCD	Distance between the buccal and lingual cervices	All teeth
Buccal dentine horn height	BDH	Distance between the buccal dentine horn and BCD	Unworn only
Lingual dentine horn height	LDH	Distance between the lingual dentine horn and BCD	Unworn only
Occlusal basin dentine diameter	OB Diameter	Dentine width along a line tangent to the lowest point of the occlusal basin at the EDJ	All teeth
Mid dentine diameter	Mid Diameter	Dentine width along a line equidistant between OB diameter and BCD	All teeth
Linear enamel thickness			
Linear Buccal Tip	LBT	Distance between the buccal cusp tip and dentine horn	Unworn only
Linear Lingual Tip	LLT	Distance between the lingual cusp tip and dentine horn	Unworn only
Linear Occlusal Basin	LOB	Distance between the lowest point of the enamel in the occlusal basin and underlying EDJ	Unworn only
Average enamel thickness			
Enamel‐dentine junction	EDJ	Length of the boundary between enamel and dentine	All teeth
Average enamel thickness	AET	Area of the enamel cap divided by EDJ	All teeth
Relative enamel thickness	RET	AET divided by dentine area multiplied by 100	All teeth
Regional enamel thickness			
Buccal lateral wall thickness	BLAET	Enamel area between buccal cusp tip and buccal CEJ divided by the length of the EDJ between the same two points	Unworn only
Lingual lateral wall thickness	LLAET	Enamel area between lingual cusp tip and buccal CEJ divided by the length of the EDJ between the same two points	Unworn only
Buccal occlusal basin thickness	BOAET	Enamel area between buccal cusp tip and LOB divided by the length of the EDJ between the same two points	Unworn only
Lingual occlusal basin thickness	LOAET	Enamel area between lingual cusp tip and LOB divided by the length of the EDJ between the same two points	Unworn only

### 2.2 Measurements

**FIGURE 2:**
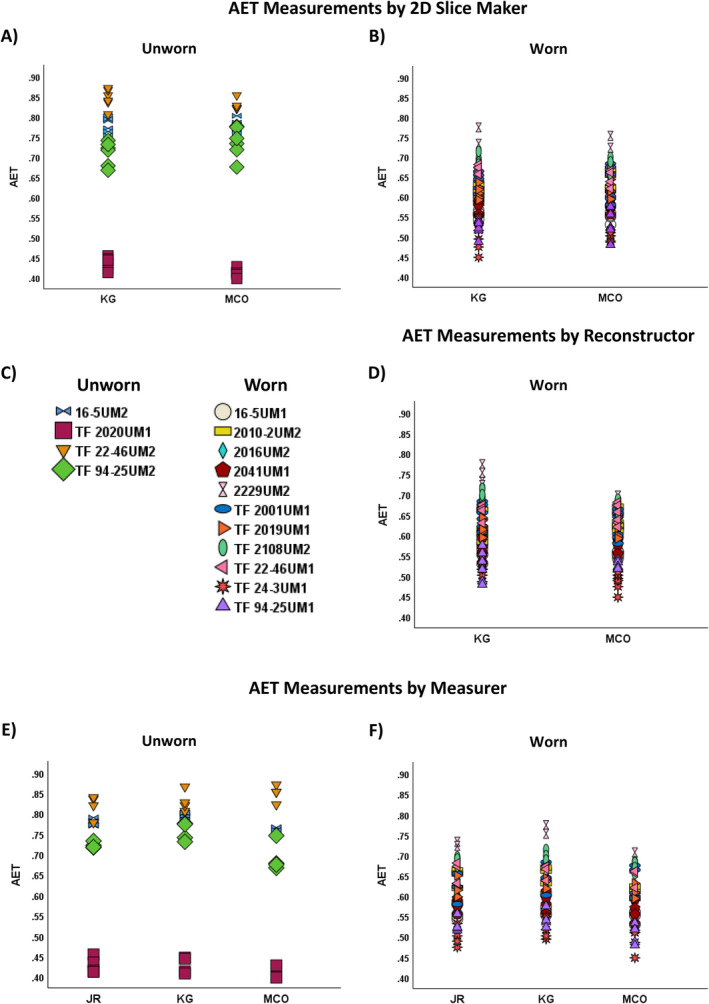
AET measurements, organized by who made the 2D slice (A, B), who did the reconstruction (D), and who did tooth the measurement (E, F). Each tooth is represented on the legend (C).

The measurements included in this study were those we considered most likely to introduce human error at different points in the process of measuring teeth using μCT scans and that are most frequently used by dental anthropologists (Table 2). The angle at which the 2D slice is taken relative to the cervical margin can impact the virtual render of the tooth (Figure 2), subsequently affecting standard measurements like the bicervical diameter (BCD) (Benazzi et al. 2014). Additionally, we evaluated measurement of dentine horn heights and dentine diameters to capture the slight variation in shape that can be produced by slight differences in the occlusal‐cervical slice angle (Figure 2; Table 2; Smith et al. 2006). A variety of linear enamel thickness measurements are taken by dental anthropologists, and we have included three of the most common (Table 2; Macho and Thackeray 1993). Whole‐crown summaries of enamel thickness are recorded using the standard AET and RET from Martin (1985) for both worn and unworn teeth. Regional enamel thicknesses were also measured only on unworn teeth (Kono and Suwa 2008; Skinner et al. 2015). Error analyses were completed for both worn and unworn teeth to assess the impact of wear on error generation. However, certain measurements require unworn teeth and could therefore not be taken on digitally reconstructed sections. Consequently, for reconstructed teeth only, the following measurements were recorded: BCD, EDJ. Cap area, Dentine area, OB diameter, Mid diameter, Crown area, AET, and RET (see O'Hara and Guatelli‐Steinberg 2022; Table 2).

### 2.3 Statistical Methods

Inter‐observer percent error was calculated using SPSS v.22 three times per slice once for each pair of measures created by the different observers (J.R. vs. K.N.G., K.N.G. vs. M.C.O., J.R. vs. M.C.O.) as $\left[\frac{\left|\text{measurement}\;1-\text{measurement}\;2\right|}{\left[\frac{\text{measurement}\;1+\text{measurement}\;2}{2}\right]}*100\right]$. The three resulting numbers were then averaged to produce an overall relative inter‐observer percent error mean (Popovic and Thomas 2017) and a standard deviation was calculated. Percent errors for worn and unworn teeth were calculated separately to account for possible additive error when worn teeth were reconstructed. For measurements that averaged percent errors > 5%, or for those with means of one standard deviation of 5%, univariate ANOVAs were calculated to evaluate relative variance from slice maker, reconstructor, tooth, and measurer.

## 3 Results

### 3.1 Unworn Teeth

For unworn teeth, six of the 14 measurements had total average errors > 5% (Table 3). These “high error” measurements were LLT, LOB, LBT, LOAET, BOAET, and BLAET. Additionally, AET, RET, and LLAET had mean ±1 SD errors that exceeded 5%. All linear enamel thickness measurements (LBT, LLT, and LOB) produced errors > 5%. These measurements only range from 0.14 to 1.4 mm and can be heavily affected by placement of the cusp tip for measurement, a decision made by the individual measurer. In turn, LOAET, BOAET, and BLAET are area measurements determined by the placement of the linear measurements and represent only small portions of the tooth.

**TABLE 3:** Mean and standard deviation (SD) of total percent error for unworn teeth. Bold and shaded cells indicate an average error greater than 5%.

	Mean	SD
BCD	0.91	0.89
BDH	2.04	1.56
LDH	1.80	1.55
OB Diameter	1.51	1.23
Mid Diameter	2.04	2.06
LBT	**16.14**	13.86
LLT	**26.50**	30.05
LOB	**11.75**	9.00
AET	3.46	2.35
RET	4.34	2.57
BLAET	**5.84**	5.27
LLAET	4.46	4.00
BOAET	**8.13**	6.70
LOAET	**10.07**	8.02

Table 4 reports the ANOVA results for the measurements with error ranges > 5%. Every model was significant and, in every case, the tooth measured was a significant source of variation. The person producing the 2D slices (K.N.G., M.C.O.) was never a significant source of variation. Measurer was a statistically significant source of variation for LLT, LOB, AET, and BOAET for the ANOVA (Table 4).

**TABLE 4:** ANOVAs for unreconstructed (unworn) Teeth ONLY. Bold and highlighted cells show a statistically significant result (*p* < 0.05).

	Intercept	Tooth	Slice maker	Measurer
LBT	**0.008**	**< 0.001**	0.363	0.529
LLT	**0.028**	**< 0.001**	0.728	**0.016**
LOB	**0.004**	**< 0.001**	0.779	**< 0.001**
AET	**0.005**	**< 0.001**	0.421	**0.033**
RET	**0.001**	**< 0.001**	0.093	0.211
BLAET	**0.002**	**< 0.001**	0.490	**0.003**
LLAET	**0.009**	**< 0.001**	0.265	0.478
BOAET	**0.007**	**< 0.001**	0.104	**0.005**
LOAET	**0.002**	**< 0.001**	0.137	0.058

### 3.2 Worn Teeth

For worn teeth, there were no mean percent errors that averaged more than 5% (Table 5). However, AET and RET mean ±1 SD exceeded 5%, so they were evaluated with an ANOVA to identify the source of variation (Table 6). For both AET and RET, the tooth measured, the person creating the reconstruction, and the person performing the measurement were significant sources of variation (Table 6). Both AET and RET are equations that include several measurements that contribute to the final value, potentially creating additive error (Table 2).

**TABLE 5:** Mean and standard deviation (SD) of total percent error for worn teeth.

	Mean	SD
BCD	0.79	1.41
OB Diameter	1.14	1.25
Mid Diameter	1.56	1.90
EDJ	1.46	2.10
AET	4.28	3.46
RET	4.65	2.75

**TABLE 6:** ANOVAs for RECONSTRUCTED (Worn) Teeth ONLY. Bold and shaded cells indicate a statistically significant result (*p* < 0.05).

Metric	Intercept	Tooth	Slice maker	Reconstructor	Measurer
AET	**< 0.001**	**< 0.001**	0.924	**0.003**	**< 0.001**
RET	**< 0.001**	**< 0.001**	0.625	**0.010**	**< 0.001**

## 4 Discussion

We found that measurement error was usually low (< 5%) regardless of whether a tooth was worn (with the exception of small linear measurements like those at the cusp tip). In general, our results indicate that μCT tooth scans can be sliced, reconstructed, and measured by multiple individuals without significant inter‐observer error.

### 4.1 Linear Measurements Are Prone to Error

The linear measures of enamel thickness (LLT, LOB, and LBT) were only measured on unworn teeth. Similar to O'Hara and Guatelli‐Steinberg (2022), we found that these small linear measurements were prone to high absolute percent error, often exceeding 10% or 15%. Also like these authors, we suggest that this “high error” arises because these measurements are relatively small, generally less than 1 mm. In these instances, “every pixel count” on the screen and extremely slight variations in measurement position can lead to high percent error even if the absolute difference is only 0.02 mm, for example. In many cases, LLT, LOB, and LBT measured under 1 mm or even under 0.5 mm. With measurements this small, even minute differences (e.g., 0.95 mm vs. 1.01 mm) can result in high percent error (O'Hara et al. 2019). Overall, the highest inter‐observer error was 26.50% at the LLT of the lingual cusp tip. Measurements at the buccal cusp tip (LBT) were similarly high at 16.14%. We caution that error may be high when measuring cusp tips in part because of variation in where the cusp tip is determined to be.

### 4.2 Wear Does Not Impact Scan Shareability

Total average percent error did not exceed an average of 5% for any of the measurements performed on worn teeth. This is interesting, considering that the reconstruction of the worn teeth could have changed the shape of the crown which may have affected measurements of dentine area, enamel cap area, and EDJ, as well as compound metrics that rely on measurements of shape like AET and RET. The fact that there was no error greater than 5% suggests that at least for measurement error, worn and reconstructed teeth are not problematic, at least for measurements examined in this study.

For worn teeth, making 2D slices is more difficult, as the dentine horns are worn away and reconstruction of cusp tips only occurs after slice‐making. The ANOVA results (Table 6) indicate that different people can generate similar 2D slices even when a tooth is worn, and that inter‐observer variation is more problematic for the act of reconstruction. Figure 2 shows the complete range of variation in AET measurements produced by tooth, 2D slice‐maker, reconstructor, and measurer. Even though reconstructor and measurer are sources of significant variation for AET and RET, the range and magnitude of difference in measurement is negligible (Tables 3, 4, 5, 6 and Figure 2).

### 4.3 Sources of Measurement Variation

Regarding the source of variation in measurements, the tooth itself was always a significant factor, while the person creating the 2D slice was never significant (Figure 2). This suggests that multiple individuals can reliably generate comparable 2D slices from the same TIFF stack. For worn teeth, the reconstructor was a statistically significant source of variation for AET and RET. This is understandable as Crown Area is a factor in the equation that produces both AET and RET (Table 2). We caution against the use of many different reconstructors or reconstruction methods if AET or RET will be calculated; however, the range and magnitude of the actual differences in measurement is small (Figure 2, D).

## 5 Conclusion

The sharing of μCT scans can make research more transparent and accessible, facilitate collaboration, and enhance the practice of open research and the interconnectedness of science. In this paper, we found minor differences in measurement due to 2D slice maker, reconstructor, and measurer. For the majority of the measurements examined in this study, comparable results can be achieved when different people reconstruct, slice, and measure μCT scans of teeth. Because worn teeth do not always have dentine horns to help guide the position of the 2D slices, extra attention and consideration should be taken on the part of the researcher when creating the 2D slice. Even without the guidance of the dentine horn position, our results are still comparable between slices. We also caution that very small linear measurements are prone to high percent measurement error. However, there is no indication that total average inter‐observer error increases with each additive image process (2D slice creation, reconstruction, etc.). Across the board, measurement differences were minimal, even considering the tooth, 2D slice maker, wear reconstructor, and measurer.

## Author Contributions

**Kaita N. Gurian:** conceptualization (equal), data curation (equal), formal analysis (equal), investigation (lead), methodology (equal), supervision (equal), validation (equal), visualization (equal), writing – original draft (lead), writing – review and editing (equal). **Debra Guatelli‐Steinberg:** funding acquisition (equal), writing – review and editing (equal). **W. Scott McGraw:** funding acquisition (equal), writing – review and editing (equal). **Jess Rychel:** data curation (supporting), investigation (supporting). **Mackie C. O'Hara:** conceptualization (equal), data curation (equal), formal analysis (equal), investigation (equal), methodology (equal), supervision (equal), validation (equal), visualization (equal), writing – original draft (equal), writing – review and editing (equal).

## Supporting information


**Data S1** Supporting Information.

## Data Availability

Data that support the findings of this study are available as a Supporting Information.

## References

[ajpa70001-bib-0001] Bailey, S. E. , S. Benazzi , and J. J. Hublin . 2014. “Allometry, Merism, and Tooth Shape of the Upper Deciduous M2 and Permanent M1.” American Journal of Physical Anthropology 154, no. 1: 104–114. 10.1002/ajpa.22477. 24482249

[ajpa70001-bib-0002] Benazzi, S. , M. Fantini , F. De Crescenzio , F. Persiani , and G. Gruppioni . 2009. “Improving the Spatial Orientation of Human Teeth Using a Virtual 3D Approach.” Journal of Human Evolution 56, no. 3: 286–293. 10.1016/j.jhevol.2008.07.006. 19167741

[ajpa70001-bib-0003] Benazzi, S. , D. Panetta , C. Fornai , M. Toussaint , G. Gruppioni , and J. J. Hublin . 2014. “Technical Note: Guidelines for the Digital Computation of 2D and 3D Enamel Thickness in Hominoid Teeth.” American Journal of Physical Anthropology 153, no. 2: 305–313. 10.1002/ajpa.22421. 24242830

[ajpa70001-bib-0005] Kono, R. T. , and G. Suwa . 2008. “Enamel Distribution Patterns of Extant Human and Hominoid Molars: Occlusal Versus Lateral Enamel Thickness.” Bulletin of the National Museum of Nature and Science D 34: 1–9.

[ajpa70001-bib-0006] Macho, G. A. , and J. F. Thackeray . 1993. “Computed Tomography and Intercuspal Angulation of Maxillary Molars of Plio‐Pleistocene Hominids From Sterkfontein, Swartkrans and Kromdraai (South Africa): An Exploratory Study.” Zeitschrift für Morphologie und Anthropologie 79, no. 3: 261–269. 10.1127/zma/79/1992/261. 8128755

[ajpa70001-bib-0007] Martin, L. 1985. “Significance of Enamel Thickness in Hominoid Evolution.” Nature 314, no. 6008: 260–263. 10.1038/314260a0. 3920525

[ajpa70001-bib-0008] Martin, L. B. , A. J. Olejniczak , and M. C. Maas . 2003. “Enamel Thickness and Microstructure in Pitheciin Primates, With Comments on Dietary Adaptations of the Middle Miocene Hominoid Kenyapithecus.” Journal of Human Evolution 45, no. 5: 351–367. 10.1016/j.jhevol.2003.08.005. 14624746

[ajpa70001-bib-0009] Molnar, S. 1971. “Human Tooth Wear, Tooth Function, and Cultural Variability.” American Journal of Physical Anthropology 34: 175–190. 5572602 10.1002/ajpa.1330340204

[ajpa70001-bib-0010] Molnar, S. , and D. G. Gantt . 1977. “Functional Implications of Primate Enamel Thickness.” American Journal of Physical Anthropology 46, no. 3: 447–454. 10.1002/ajpa.1330460310. 404884

[ajpa70001-bib-0011] O'Hara, M. C. , and D. Guatelli‐Steinberg . 2022. “Reconstructing Tooth Crown Heights and Enamel Caps: A Comparative Test of Three Existing Methods With Recommendations for Their Use.” Anatomical Record 305, no. 1: 123–143. 10.1002/ar.24637. 33843152

[ajpa70001-bib-0012] O'Hara, M. C. , A. Le Cabec , S. Xing , M. F. Skinner , and D. Guatelli‐Steinberg . 2019. “Safe Casting and Reliable Cusp Reconstruction Assisted by Micro‐Computed Tomographic Scans of Fossil Teeth.” Anatomical Record 302, no. 9: 1516–1535. 10.1002/ar.24047. 30537229

[ajpa70001-bib-0013] Popovic, Z. B. , and J. D. Thomas . 2017. “Assessing Observer Variability: A User's Guide.” Cardiovascular Diagnosis and Therapy 7, no. 3: 317–324. 10.21037/cdt.2017.03.12. 28567357 PMC5440257

[ajpa70001-bib-0014] Skinner, M. M. , Z. Alemseged , C. Gaunitz , and J. J. Hublin . 2015. “Enamel Thickness Trends in Plio‐Pleistocene Hominin Mandibular Molars.” Journal of Human Evolution 85: 35–45. 10.1016/j.jhevol.2015.03.012. 26024565

[ajpa70001-bib-0015] Skinner, M. M. , P. Gunz , B. A. Wood , and J. J. Hublin . 2008b. “Enamel‐Dentine Junction (EDJ) Morphology Distinguishes the Lower Molars of *Australopithecus africanus* and *Paranthropus robustus* .” Journal of Human Evolution 55, no. 6: 979–988. 10.1016/j.jhevol.2008.08.013. 18824253

[ajpa70001-bib-0016] Skinner, M. M. , B. A. Wood , C. Boesch , et al. 2008a. “Dental Trait Expression at the Enamel‐Dentine Junction of Lower Molars in Extant and Fossil Hominoids.” Journal of Human Evolution 54, no. 2: 173–186. 10.1016/j.jhevol.2007.09.012. 18048081

[ajpa70001-bib-0017] Smith, T. M. , A. J. Olejniczak , K. Kupczik , et al. 2009. “Taxonomic Assessment of the Trinil Molars Using Non‐Destructive 3D Structural and Development Analysis.” Paleoanthropology 2009: 117–129.

[ajpa70001-bib-0018] Smith, T. M. , A. J. Olejniczak , D. J. Reid , R. J. Ferrell , and J. J. Hublin . 2006. “Modern Human Molar Enamel Thickness and Enamel–Dentine Junction Shape.” Archives of Oral Biology 51, no. 11: 974–995. 16814245 10.1016/j.archoralbio.2006.04.012

